# Additions to hyphomycetes from Yungui Plateau, China with three new species (Ascomycota, Sordariomycetes)

**DOI:** 10.3897/BDJ.11.e101629

**Published:** 2023-05-15

**Authors:** Long Chun-Sheng, Wu You-Peng, Zhang Xu, Lin Yan, Shen Xiang-Chun, Ma Jian, LI Qi-Rui

**Affiliations:** 1 State Key Laboratory of Functions and Applications of Medicinal Plants, Guizhou Medical University, Guiyang, China State Key Laboratory of Functions and Applications of Medicinal Plants, Guizhou Medical University Guiyang China; 2 The Key Laboratory of Optimal Utilization of Natural Medicine Resources, School of Pharmaceutical Sciences, Guizhou Medical University, Guiyang, China The Key Laboratory of Optimal Utilization of Natural Medicine Resources, School of Pharmaceutical Sciences, Guizhou Medical University Guiyang China; 3 The High Educational Key Laboratory of Guizhou Province for Natural Medicinal Pharmacology and Druggability, School of Pharmaceutical Sciences, Guizhou Medical University, University Town, Guiyang, China The High Educational Key Laboratory of Guizhou Province for Natural Medicinal Pharmacology and Druggability, School of Pharmaceutical Sciences, Guizhou Medical University, University Town Guiyang China; 4 The Union Key Laboratory of Guiyang City-Guizhou Medical University, School of Pharmaceutical Sciences, Guizhou Medical University, University Town, Guiyang, China The Union Key Laboratory of Guiyang City-Guizhou Medical University, School of Pharmaceutical Sciences, Guizhou Medical University, University Town Guiyang China; 5 College of Agronomy, Jiangxi Agricultural University, Nanchang, China College of Agronomy, Jiangxi Agricultural University Nanchang China

**Keywords:** Guizhou, karst area, new taxa, phylogenetic analysis, taxonomy

## Abstract

**Background:**

Yungui Plateau is rich in fungal diversity. Hyphomycetes, growing on submerged wood, can promote the degradation of organisms and the reuse of rotten wood energy. During an investigation of hyphomycetes in this region, 19 species of dematiaceous hyphomycetes were collected in Yungui Plateau.

**New information:**

Both morphological identification and multi-gene phylogenetic analyses of ITS, *tef*1 and LSU sequences supported *Coryneumsevenseptatis* as a new species. *Phaeoisariaguizhouensis* and *Pleurotheciumyunanensis* were introduced, based on morphology. Morphological descriptions and illustrations of the new species were detailed. Known species are listed with notes.

## Introduction

*Coryneum* firstly described by Nees based on the *C.umbonatum* Nees, 1816 (Nees von Esenbeck 1816). Some synapomorphies between Coryneaceae and Pseudovalsaceae like having black perithecia, often immersed in wood, asci that deliquesce at maturity and an asexual morph with transversely distoseptate brown conidia, are regarded as the character of Coryneaceae and many species of this genus have been reported as phytopathogens causing tree canker (Sutton 1975, Rossman et al. 2007, Senanayake et al. 2017). In the recent decade, tens of species of *Coryneum* have been reported, and five species have been found in China (Rossman et al. 2015, Senanayake et al. 2017, Jiang et al. 2018, Senwanna et al. 2018, Jiang et al. 2019, Boonmee et al. 2021).

*Phaeoisaria* was firstly described by Höhnel (1909) to accommodate *P.bambusae* Höhn., 1909 as the type species. This genus is characterised by conidiophores adpressed in parallel with numerous sympodially extending denticulate conidiogenous cells and aseptate or septate ellipsoidal, obovoidal, fusiform-cylindrical to falcate, hyaline conidia (Höhnel 1909, Hyde et al. 2018, Boonyuen et al. 2021) In the recent decade, increasing numbers of species of *Phaeoisaria* have been considered as new species (Crous et al. 2017, Hyde et al. 2018, Luo et al. 2019, Boonmee et al. 2021, Crous et al. 2021, Liu et al. 2022, Jayawardena et al. 2023).

*Pleurothecium* was firstly described by Höhnel (1919) to accommodate *P.recurvatum*as (Morgan) Höhn. the type species. This genus is characterised by distinct brown conidiophores and polyblastic sympodially extended denticulate conidiogenous cells. The conidia are solitary, unicellular or septate, hyaline or pigmented (Matsushima 1975, Matsushima 1980, Cooper 2005, Réblová et al. 2012). In the recent decade, five species of *Pleurothecium* have been reported (Monteiro et al. 2016, Hyde et al. 2017, Luo et al. 2018,Shi et al. 2021, Jayawardena et al. 2023).

Yungui Plateau is a typical karst landform including Guizhou and Yunnan Provinces in south-western China (Wang et al. 2004). Guizhou Province is located in the east of Yungui Plateau. Its warm climate has led to the development of various subtropical flora in this region. In the high-temperature area of the low latitude valley in the south, there are tropical elements and near-tropical vegetation types (Wu 2000). The environmental and biological factors resulting from the complicated geography and topography, highly variable climate conditions, diversified vegetation and forest type etc. provide an abundant and wide variety of favourable habitats and symbiotic hosts for the growth and reproduction of the fungi such as hyphomycetes (Wu 2000, Gulis and Suberkropp 2003, Wu and Zhuang 2008, Wijayawardene et al. 2021). Hyphomycetous taxa are mainly saprobic on plant residues, such as rotten wood, dead branches, bark and fallen leaves, as well as in soil and submerged substrates of freshwater (Pirozynski and Hodges 1973, Gönczöl and Révay 2003, Shirouzu and Harada 2008, Shirouzu et al. 2010, Ma 2012, Yen et al. 2012, Ma et al. 2015) and have a strong ability to degrade wood fibres (Shearer and Webster 1991, Hoffmann and Hering 2000, Zhang 2018). Amongst them, hyphomycetes growing on rotten wood account for a large part. At present, more than 1400 genera of asexual hyphomycetes have been recognised all over the world (Seifert and Gams 2011). Fungal diversity in Yungui Plateau is high, but still mostly unexplored. During an investigation of hyphomycetes associated with plant residues in this region, nineteen species were identified from rotting wood, including three new species that are described below.

## Materials and methods

### Sample collection and isolation

Decaying wood with fungi were collected from forests in Guizhou and Yunnan Provinces. The samples were placed in paper bags and brought to the laboratory. Specimens were examined using an OLYMPUS SZ6 dissecting microscope. Photomicrographs were taken using a Cannon EOS 700D camera attached to a Nikon ECLIPSE Ni compound microscope. Measurements were made using the Tarosoft (R) Image Frame Work programme. Dimensions of anatomical features were shown followed Kurniawati et al. (2010). Figures were processed with Adobe Photoshop CS6 software (Adobe Systems. USA) without modification of morphological characteristics (Dong et al. 2020). Singer spore isolations were used for obtaining pure cultures (Choi et al. 1999). Germinating conidia with a small amount of medium were individually transferred to potato dextrose agar (PDA) medium plates under a stereomicroscope (Luo et al. 2018). Specimens were deposited at the Herbarium of Guizhou Medical University (GMB) and the Herbarium of Kunming Institute of Botany (KUN). The cultures were preserved in Guizhou Medical University Culture Collection (GMBC) (Table 1).

### DNA extraction, polymerase chain reaction (PCR) amplification and sequencing

The mycelia cultured on PDA medium were scraped out and put into a 1.5 ml centrifuge tube for DNA extraction. DNA Extraction Kit (E.Z.N.A.® Forensic DNA Kit, D3591, BIOMEGA, USA) was used for extraction of total DNA following its instructions. Three gene regions were amplified with universal primers. ITS1/ITS4 for the internal transcribed spacer regions of ribosomal DNA (ITS) (White et al. 1990), LR0R/LR5 for the large subunit nuclear ribosomal DNA (LSU) (Vilgalys and Hester 1990), EF1-983F/EF1-2218R for the translation elongation factor 1-alpha gene (*tef*) (Rehner and Buckley 2005, Hosaka et al. 2006) were used for PCR amplification. The PCR amplification solution included 12.5 μl of 2 × Taq PCR master mix, 9.5 μl of ddH_2_O, 1 μl of DNA extraction, 1 μl of forward primer and 1 μl of reverse primer. The PCR products were sent to Sangon Biotech, Shanghai, China, for sequencing.

### Phylogenetic analyses

**Figure 1.**
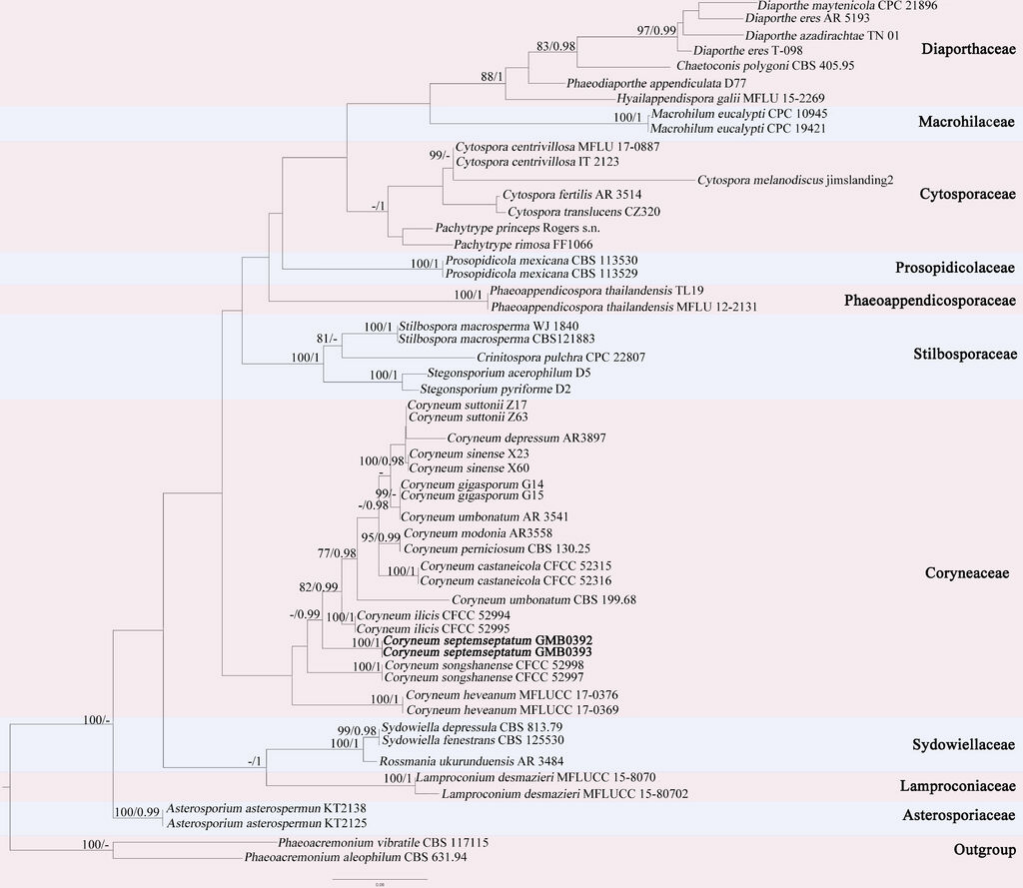
The Maximum Likelihood (RAxML) tree, based on a combined dataset of ITS, LSU and *tef*1 sequences. Bootstrap support values for Maximum Likelihood (ML, left) equal to or greater than 75% and Bayesian posterior probabilities (BY, right), equal to or greater than 0.95, are indicated at the nodes. When the value is less than 75/0.95, the value is represented by -/-. The tree is rooted to *Phaeoacremoniumvibratile* (CBS 117115) and *Phaeoacremoniumaleophilum* (CBS 631.94). The new collections are in bold. MH780882 (tef sequence of *Coryneumheveanum*) cannot be used in Phylogenetic analyses due to the abnormal Phylogenetic tree.

The sequences were pasted into the BLASTN for preliminary identification. All sequences were selected, based on the top hits and the latest literature (Boonyuen et al. 2021). The sequences were aligned with the Multiple Sequence Alignment Programme MAFFT (MAFFT 7.205) software (Katoh and Standley 2013). Then spurious sequences or poorly-aligned regions from a multiple sequence alignment were removed with the tool of TrimAl (Capella-Gutiérrez et al. 2009). Alignment Transformation EnviRonment (http://www.sing–group.org/alter/) was performed to convert the FASTA format to the phylp format (Glez-Peña et al. 2010). The Maximum Likelihood analysis was carried out with GTR+G+I model of site substitution by using RAxML 7.4.2 black box (https://www.phylo.org/, Stamatakis et al. (2008)). Bayesian analysis was performer with MrBayes v. 3.1.2 (Huelsenbeck and Ronquist 2001). The branch support was evaluated with a bootstrapping method of 1000 replicates (Hillis and Bull 1993). Posterior probabilities (PP) were determined by Markov Chain Monte Carlo sampling (MCMC) in MrBayes v. 3.2.2 (Ronquist et al. 2012). The nucleotide substitution model was estimated by MrModelTest v.2.3 (Posada and Crandall 1998). Six simultaneous Markov chains were run for 2,000,000 generations and the trees were sampled each 100^th^ generation. The first 25% of trees, greater than 0.95, are indicated at the nodes. When the value is less than 75/0.95, the value is represented by -/-. The tree is rooted to *Phaeoacremoniumaleophilum* (CBS 631.94) and *P.vibratile* (CBS 117115). The new collections are in bold (Fig. 1).

## Taxon treatments

### Coryneum septemseptatum

C.S. Long, Q.R. Li & Jian Ma sp. nov.

1B9AA989-B499-55C3-9701-9D06714BDF43

844574

#### Materials

- **Type status:** Holotype. **Occurrence:** recordedBy: Chun-Sheng Long; occurrenceID: 15C96932-06C5-5908-8A9A-309902D78913; **Taxon:** scientificName: *Coryneumseptemseptatum*; acceptedNameUsage: *Coryneumseptemseptatum* C.S. Long, Q.R. Li & Jian Ma, 2021, sp. nov; parentNameUsage: Coryneum Nees 1816; kingdom: fungi; phylum: Ascomycota; class: Sordariomycetes; order: Diaporthales; family: Coryneaceae; genus: Coryneaceae; taxonRank: species; verbatimTaxonRank: species; scientificNameAuthorship: C.S. Long, Q.R. Li & Jian Ma; **Location:** continent: Asia; country: China; stateProvince: Guizhou; county: Luodian county; locality: Daxiaojing Forest Park; verbatimElevation: 562 m; locationRemarks: label transliteration："Guizhou, Daxiaojing Forest Park，2021.10.21,Long chun-sheng"[贵州罗甸县大小井森林公园，2021年10月21日，龙春升]; verbatimCoordinates: 23.2328N, 101.2364E; georeferenceProtocol: label; **Identification:** identifiedBy: Chun-Sheng Long, Qi-Rui Li & Jian Ma; **Event:** samplingProtocol: collecting; eventDate: 10/21/2021; habitat: decaying wood; **Record Level:** type: PhysicalObject; language: en; institutionID: KUN-HKAS 12345; collectionID: GMB0392; institutionCode: The Herbarium of Cryptogams Kunming Institute of Botany AcademiaSinica
- **Type status:** Other material. **Occurrence:** occurrenceID: 3ADA3585-EE9E-58A8-882A-ADDAA0B36DA2; **Taxon:** scientificName: *Coryneumseptemseptatum*; **Location:** continent: Asia; country: China; stateProvince: Guizhou; county: Luodian county; locality: Daxiaojing Forest Park; verbatimElevation: 544 m; verbatimCoordinates: 23.8287°N, 101.4264°E; **Record Level:** type: PhysicalObject; institutionID: KUN-HKAS 12345; collectionID: GMBC0393; institutionCode: The Herbarium of Cryptogams Kunming Institute of Botany Academia Sinica

#### Description

**Figure 2.**
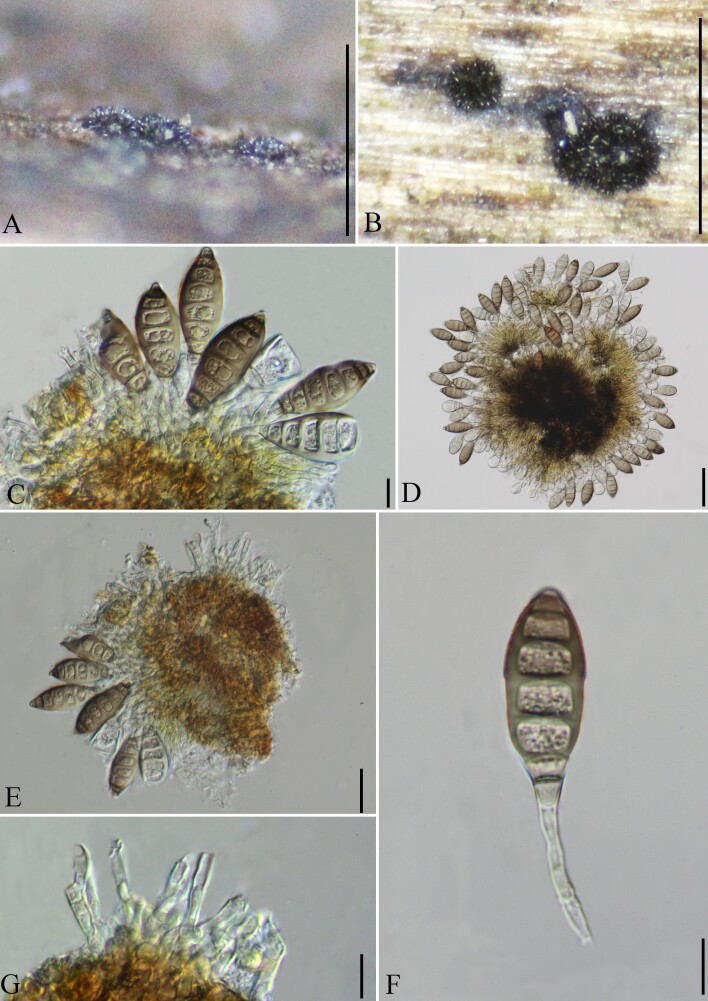
*Coryneumseptemseptatum* (holotype, GMB0392). **A, B** Conidiomata on the natural substratum; **C–F** Conidiogenous cells and Conidia; **G** Conidiophores. Scale bars: A, B = 0.5 mm, C, F, G = 10 μm, D = 50 μm, E = 25 μm.

Saprobic on the surface of decaying wood (Fig. 2). **Asexual morph**: Colonies on natural substratum effuse, brown, hairy. Mycelium superficial and immersed, composed of branched, septate, pale brown to brown, smooth-walled hyphae. Sporodochia on natural subtrate scattered or clustered, punctiform, dark brown, 77–99 µm wide, 27–44 µm high. Conidiophores micronematous or semi-macronematous, simple, 0–3-septate, pale brown, 12–31 µm long (x̅ = 20 μm, SD = 4.8, n = 15), 2.5–4 µm wide (x̅ = 3.1 μm, SD = 0.5, n = 15). Conidiogenous cells holoblastic, integrated, terminal, indeterminate, cylindrical and hyaline, with 0–1 percurrent extensions. Conidia solitary, acrogenous, dry, ellipsoidal to broadly fusiform, 34–46 μm long (x̅ = 40 μm, SD = 3.7, n = 20), 14.5–17 μm wide (x̅ = 15.5 μm, SD = 0.9, n = 20), 7–8-septate, smooth, brown, hyaline at the top, 4.5–8.5 μm (x̅ = 5.9 μm, SD = 1.1, n = 20) wide at the truncate base. **Sexual morph**: Undetermined.

#### Etymology

With reference to the conidia with 7–8 septa.

#### Notes

Amongst the known species of *Coryneum*, *C.betulinum* Schulzer, *C.gregoryi* B. Sutton, *C.japonicum* (Sacc.) B. Sutton and *C.psidii* B. Sutton are similar to *C.sevenseptatum* in conidial shape (Senwanna et al. 2018, Shavrin and Smetana 2020). However, the conidia of *C.septemseptatum* have 7–8 septa which differ from those of *C.betulinum* (4–5-septate), *C.gregoryi* (5–9-septate), *C.japonicum* (5–7-septate) and *C.psidii* (5–6-septate). Both morphological and molecular data (Fig. 1) supported *C.septemseptatum* as a new species (Table 2).

### Phaeoisaria guizhouensis

C.S. Long, Q.R. Li & Jian Ma sp. nov.

ED3AF6C1-38FB-55EF-9B9A-D577689A96B2

844575

#### Materials

- **Type status:** Holotype. **Occurrence:** recordedBy: Chun-Sheng Long; occurrenceID: 18602EC9-B7A5-5621-B1D0-ABE14D58CE17; **Taxon:** scientificName: *Phaeoisariaguizhouensis*; acceptedNameUsage: *Phaeoisariaguizhouensis* C.S. Long, Q.R. Li & Jian Ma, 2021, sp. nov.; parentNameUsage: Phaeoisaria Höhn. 1909; kingdom: fungi; phylum: Ascomycota; class: Sordariomycetes; order: Pleurotheciales; family: Pleurotheciaceae; verbatimTaxonRank: species; scientificNameAuthorship: C.S. Long, Q.R. Li & Jian Ma; taxonRemarks: species; **Location:** continent: Asia; country: china; stateProvince: Guizhou; county: Luodian County; locality: Daxiaojing Forest Park; verbatimElevation: 653 m; locationRemarks: label transliteration:"Guizhou, Daxiaojing Forest Park, 2021.10.21,Long chun-sheng";[贵州罗甸县大小井森林公园，2021年10月21日]; verbatimCoordinates: 23.5663N, 101.3213E; georeferenceProtocol: Lable; **Identification:** identifiedBy: Chun-Sheng Long, Qi-Rui Li & Jian Ma; **Event:** samplingProtocol: collecting; eventDate: //2021; habitat: decaying wood; **Record Level:** language: en; institutionID: KUN-HKAS 12346; collectionID: GMB0394; institutionCode: The Herbariumof Cryptogams Kunming Institute of Botany Academia Sinica
- **Type status:** Other material. **Occurrence:** occurrenceID: 58591A4C-D09E-5853-BF69-2AA3AD86987E; **Taxon:** scientificName: *Phaeoisariaguizhouensis*; **Location:** continent: Asia; country: china; stateProvince: Guizhou; county: Luodian county; locality: Daxiaojing Forest Park; verbatimElevation: 541 m; verbatimCoordinates: 23.5667°N, 101.3032°E; **Record Level:** collectionID: GMB0394

#### Description

**Figure 3.**
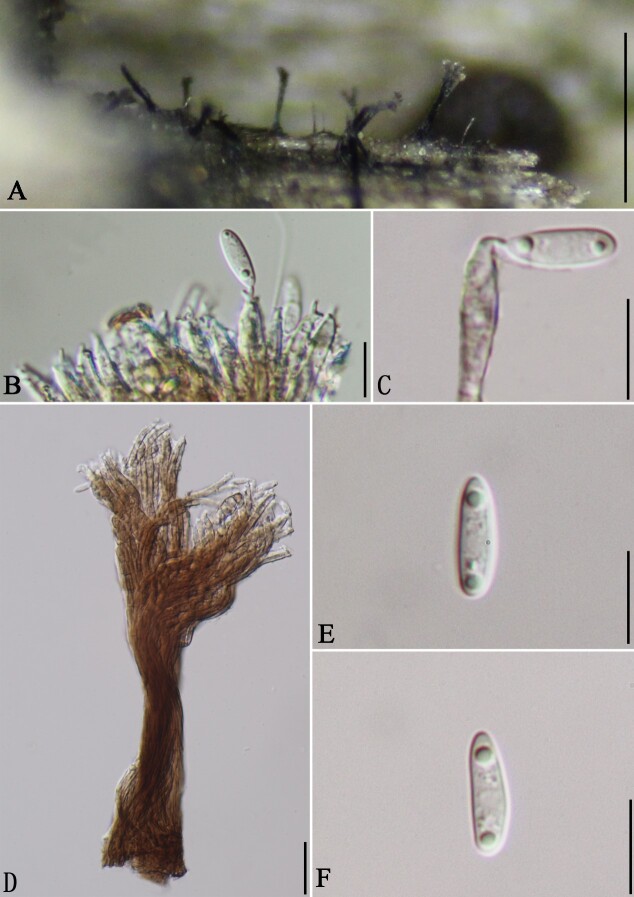
*Phaeoisariaguizhouensis* (GMB0394). **A** Conidiomata on natural substratum; **B, C** Conidiogenous cells and conidia; **D** Synnema with conidiophores and conidia; **E, F** Conidia. Scale bars: A = 0.5 mm, B–F = 10 μm.

Saprobic on the surface of decaying wood (Fig. 3). **Asexual morph**: Colonies on natural substratum. Conidial secession schizolytic. Conidia solitary, acropleurogenous, 9–13 μm long (x̅ = 11.0 μm, SD = 1.9, n = 18), 1.9–3.6 μm wide (x̅ = 3.0 μm, SD = 0.6, n = 15), ellipsoidal to obovoidal, rounded at the apex, hyaline, aseptate, guttulate, smooth-walled. **Sexual morph**: Undetermined.

#### Etymology

With reference to Guizhou Province where the type specimen was found.

#### Notes

*Phaeoisariaguizhouensis* morphologically resembles *P.aquatica* Z. L. Luo et al. and *P.pseudoclematidis* D.Q. Dai & K.D. Hyde, but they differ in conidial size (6.5–7.5 × 2.5–3.5 μm for *P.aquatic*; 9–13 × 1.9–3.6 μm for *P.guizhouensis*; 5–8.5 × 3–4 μm for *P.pseudoclematidis*) and the conidial of *P.acquatica* and *P.pseudoclimatidis* appears around the conidiophores, but *P.guizouensis* only appears at the apex (Liu et al. 2015, Luo et al. 2018).

### Pleurothecium yunanensis

C.S. Long, Q.R. Li & Jian Ma sp. nov.

524BB9E7-CF29-5D32-BD71-5420E428BBF5

844576

#### Materials

- **Type status:** Holotype. **Occurrence:** occurrenceID: 65AEAD76-F2A2-565F-9174-B0DB52CDA91C; **Taxon:** scientificName: *Pleurotheciumyunanensis*; acceptedNameUsage: *Pleurotheciumyunanensis* C.S. Long, Q.R. Li & Jian Ma, 2021, sp. nov.; parentNameUsage: Pleurothecium Höhn. 1919; kingdom: Fungi; phylum: Ascomycota; class: Sordariomycetes; order: Pleurotheciales; family: Pleurotheciaceae; genus: Pleurothecium; taxonRank: species; scientificNameAuthorship: C.S. Long, Q.R. Li & Jian Ma; taxonRemarks: species; **Location:** continent: Asia; country: China; stateProvince: Yunnan; county: Nanjian county; locality: Lingbaoshan National Forest Park; verbatimElevation: 2532 m; locationRemarks: Labeltransliteration:"Nanjian County, Lingbaoshan National Forest Park,18/8/2021,Long Chun-Sheng";[云南省南涧县灵宝山国家森林公园,18/8/2021,龙春升]; verbatimCoordinates: 22.7324°N, 100.4232° E; georeferenceProtocol: label; **Identification:** identifiedBy: Chun-Sheng Long, Qi-Rui Li & Jian Ma; **Event:** samplingProtocol: collecting; eventDate: 18/8/2021; habitat: decaying wood; **Record Level:** language: en; institutionID: KUN-HKAS 12347; collectionID: GMB0396; institutionCode: KUN-HKAS 12347
- **Type status:** Holotype. **Occurrence:** occurrenceID: 4FC9ABFC-435F-533D-931D-15AD95398CFF; **Taxon:** scientificName: *Pleurotheciumyunanensis*; **Location:** continent: Asia; country: China; stateProvince: Yunnan; county: Nanjian county; locality: Lingbaoshan National Forest Park; verbatimElevation: 2567 m; verbatimCoordinates: 22.7431N 100.4334E; **Record Level:** collectionID: GMB0394

#### Description

**Figure 4.**
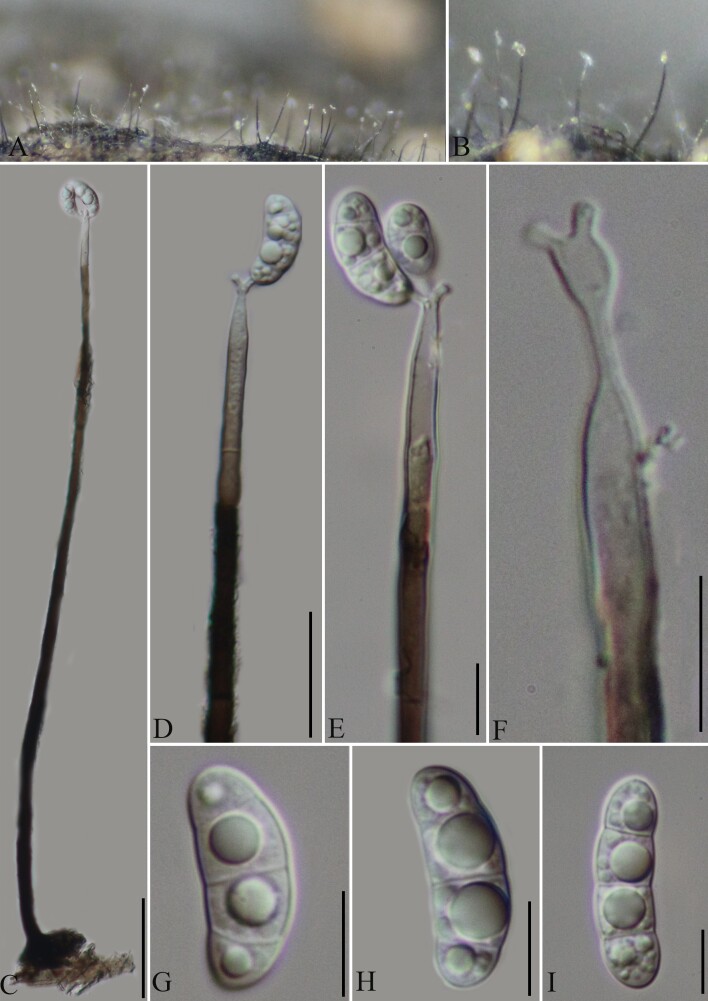
*Pleurotheciumyunnanensis* (GMB0395, holotype). **A, B** Colonies on natural substratum; **C** Conidiophore; **D, E** Conidiogenous cells and conidia; **F** Conidiogenous cells; **G−I** Conidia. Scale bars: A=0.5 mm, B = 0.5 mm, C = 50 μm, D = 30 μm, E = 15 μm, F−I =10 μm.

Saprobic on the surface of decaying wood (Fig. 4). **Asexual morph**: Conidiophores 370–206 μm long (x̅ = 270.9 μm, SD = 81.8, n = 18), 9.8–4.2 μm wide (x̅ = 6.5 μm, SD = 2.1, n = 18), mononematous, unbranched, erect, straight to slightly flexuous towards the apex, single, 4–5-septate, the lower part is black and the upper one is light brown or hyaline, smooth. Conidiogenous cells 10– 14 μm long (x̅ = 12 μm, SD = 2, n = 20), 2.5–3.5 μm wide (x̅ = 3 μm, SD = 0.5, n = 20), polyblastic, integrated, terminal, sometimes becoming intercalary, sympodially elongated, denticulate, denticles narrow cylindrical, hyaline. Conidial secession schizolytic. Conidia 17–25.6 μm long (x̅ = 22.1 μm, SD =3.6, n = 25), 2.8–9 μm wide (x̅ = 7.8 μm, SD = 1.3, n = 25), solitary, acropleurogenous, half-moon, guttulate, hyaline, 2–3-septate, smooth-walled. **Sexual morph**: Undetermined.

#### Etymology

With reference to Yunnan Province where the type specimen was found.

#### Notes

*Pleurothecium* is characterisedby the distinct brown conidiophores and polyblastic, sympodially extended, densiculate conidiogenic cells (Monteiro et al. 2016). *Pleurotheciumyunanensis* superficially resembles *P.leptospermi* J.A. Cooper and *P.pulneyense* Subram. & Bhat, but *P.leptospermi* differs by its smaller (15–18 × 4–5 μm vs. 17–25.6 × 2.8–9 μm) versicolored conidia with three eusepta (Cooper 2005); *P.pulneyense* differs by its cylindrical to fusiform, larger conidia (23–30 × 7–8.4 µm vs. 17–25.6 × 2.8–9) with three eusepta (Subramanian and Bhat 1987).

### Brachysporiella pulchra

(Subram.) S. Hughes, New Zeal. J. Bot. 17(2): 184 (1979)

06CEF810-8CF1-58BF-8E4A-5FB9CFCC3817

555675

#### Materials

- **Type status:** Other material. **Occurrence:** recordedBy: Chun-Sheng Long; occurrenceID: E1A38663-4F73-5C35-9B0B-3985BFA64030; **Taxon:** scientificName: *Brachysporiellapulchra*; **Location:** continent: Asia; country: China; stateProvince: Yunnan; county: Nanjian Yi Autonomous County; locality: Lingbaoshan National Forest Park; verbatimElevation: 2338 m; verbatimCoordinates: 24.7864N, 100.4352E; **Identification:** identifiedBy: Chun-Sheng Long, Qi-Rui Li & Jian Ma; **Event:** eventDate: 18/8/2021; habitat: on decaying wood; **Record Level:** collectionID: GMB0410

#### Description

Conidiophores 151–395 μm long (x̅ = 269.9 μm, SD = 65.7, n = 20), 3.6–6.5 μm wide (x̅ = 5 μm, SD = 1.0, n = 20), mononematous, erect, single, brown or dark brown, smooth. Conidiogenous cells 6–12 μm long (x̅ = 9.1 μm, SD = 2, n = 20), 2.5–3.5 μm wide (x̅ = 3 μm, SD = 0.5, n = 20) ampulliform to cylindrical, brown to dark brown, Conidia 15–20 μm long (x̅ = 17.3 μm, SD =1.4, n = 20), 7.9–12 μm wide (x̅ = 10 μm, SD = 1.2, n = 20), solitary, clavate, guttulate, truncated in base, hyaline, 3-septate, smooth-walled

Also see Hughes (1979).

#### Notes

*Brachysporiellapulchra* superficially resembles *B.gayana* Bat, but the conidia in *B.pulchra* are smaller (24–26 × 10.5–12.5 µm vs. 30–38 × 13–21.5 µm) (Li et al. 2019) and its conidiophores are branched. *B.pulchra* has been recorded from China, India and Japan (Gao 2016).

### Brachysporiella setosa

(Berk. & M.A. Curtis) M.B. Ellis, Mycol. Pap. 72: 17(1959)

DBE09757-AD18-5C20-A7A2-9591D54A9BF5

293842

#### Materials

- **Type status:** Other material. **Occurrence:** recordedBy: Chun-Sheng Long; occurrenceID: 8E2A7219-6507-5A85-BC9D-828B5DC7DA54; **Taxon:** scientificName: *Brachysporiellasetosa*; **Location:** continent: Asia; country: China; stateProvince: Qiannan Buyei and Miao Autonomous Prefecture; county: Libo County; locality: unknow mountain; verbatimElevation: 877 m; verbatimCoordinates: 25.1203N, 107.3243E; **Identification:** identifiedBy: Chun-Sheng Long, Qi-Rui Li & Jian Ma; **Event:** eventDate: 21/11/2021; habitat: On decaying wood; **Record Level:** collectionID: GMB0399

#### Description

Conidiophores 300–450 μm long (x̅ = 360.4 μm, SD = 30.8, n = 20), 3.6–6.5 μm wide (x̅ = 4.7 μm, SD = 0.92, n = 20), mononematous, branched in apex, erect, single, 5–7 septate, brown or dark brown, smooth. Conidiogenous cells lacking. Conidia 20–38 μm long (x̅ = 25.8 μm, SD =4.9, n = 20), 17–23 μm wide (x̅ = 18.4 μm, SD = 2.2, n = 20). Pyriform or obovoid brown or dark brown.

Also see Hughes (1958) and Ellis (1959).

#### Notes

This species was originally assigned to *Monotospora* Corda by Berk and Curtis and later was transferred to *Phragmocephala* E.W. Mason & S. Hughes, *Monosporella* S. Hughes and *Monotosporella* S. Hughes (Hughes 1958). Ellis (1959) transferred it to *Brachysporiella* Bat. as *Brachysporiellasetose*. Gao (2016) found this species in Shunhuang Mountain, Hunan Province. This fungus is mainly distributed in South Carolina and usually found on rotten wood (Sadowski et al. 2012). *B.setosa* is very close to *B.rhizoidea* (V. Rao & de Hoog) W.P. Wu in morphology, but the conidiophores of *B.setosa* are shorter (140–260 × 4.5–6 μm vs. 50–80 × 4–5 μm) (Tsui et al. 2001, Sadowski et al. 2012).

### Catenularia catenulata

(Z.L. Luo, K.D. Hyde & H.Y. Su) Réblová & A.N.Mill., MycoKeys 81: 13 (2021)

DB927775-35A8-528E-BF15-CCAE79F60D0E

839462

#### Materials

- **Type status:** Other material. **Occurrence:** recordedBy: Chun-Sheng Long; occurrenceID: A52EDDA7-0CB8-59C1-ACD4-F1651B5C5D4A; **Taxon:** scientificName: *Chaetosphaeriacatenulata*; **Location:** continent: Asia; country: China; stateProvince: Guizhou; county: Libo; locality: LantingMountain Forest Park; verbatimElevation: 866 m; verbatimLatitude: 25.1203N, 107.3431E; **Identification:** identifiedBy: Chun-Sheng Long, Qi-Rui Li & Jian Ma; **Event:** eventDate: 21/11/2021; habitat: On decaying wood; **Record Level:** collectionID: GMB0397

#### Description

Conidiophores 200–283 μm long (x̅ = 346.2 μm, SD =21.3, n = 20), 6–10 μm wide (x̅ = 7.5 μm, SD = 1.8, n = 20), cylindrical. Conidiogenous cells 21–40 μm long (x̅ = 28.5 μm, SD = 5.6, n = 20), 5.4–6.5 μm wide wide (x̅ = 6 μm, SD = 0.36, n = 20), monophialidic, integrated, terminal, cylindrical-clavate, with flared collarette. Conidia 13–15 μm long (x̅ = 14 μm, SD = 0.79, n = 20), 12–14 μm wide (x̅ = 13.2 μm, SD = 0.84, n = 20), formed in chains, aseptate, turbinate-triangular, with three blunt protruding edges at the broader distal end, hyaline to subhyaline when young, greyish-brown at maturity, smooth-walled.

Also see Réblová et al. (2021).

#### Notes

*Chaetosphaeriacatenulata* was firstly reported on submerged wood on the side of Nujiang River, Yunnan Province (Luo et al. 2019). Réblová et al. (2021) transferred it to *Catenularia* Grove as *Catenulariacatenulata*. Morphologically, it is similar to *C.cubensis* Hol.-Jech, but the latter has smaller conidia (13–15 × 12–14 μm vs. 5.5–9 × 3.5–5.5 μm) (Luo et al. 2019).

### Chloridium gonytrichii

(F.A. Fernández & Huhndorf) Réblová & Seifert, IMA Fungus 7(1): 134 (2016)

22CDBFDC-F168-57AD-B676-506AEA95B496

816827

#### Materials

- **Type status:** Other material. **Occurrence:** recordedBy: Chun-Sheng Long; occurrenceID: 619B5938-2358-5CCE-831B-AFAA7FA04C90; **Taxon:** scientificName: *Chloridiumgonytrichii*; **Location:** continent: Asia; country: China; stateProvince: Guizhou; county: Luodian; locality: Hongshui River; verbatimElevation: 39 m; verbatimCoordinates: 25.2239N, 106.5340E; **Identification:** identifiedBy: Chun-Sheng Long, Qi-Rui Li & Jian Ma; **Event:** eventDate: 18/9/2021; habitat: On decaying wood; **Record Level:** collectionID: GMB0409

#### Description

Conidiophores 190–346 μm long (x̅ = 294.1 μm, SD = 52.6, n = 20), 4.5–6.5 µm wide (x̅ = 5.5 μm, SD = 1.8, n = 20), mononematous, single, unbranched, septate, with 3-4 whorls of phialides in the mid-section and a single phialide at the apex, dark brown and paler towards the apex. Conidiogenous cells 10–14 μm long (x̅ = 12.5 μm, SD = 52.6, n = 20), 3–4 µm wide (x̅ = 5.5 μm, SD = 3.5, n = 20), cylindrical to lageniform, phialides, producing conidia from multiple entero-blastic conidiogenous loci and phialides borne on collar hyphae around the conidiophore. Conidia 3.5–4.5 μm long (x̅ = 4 μm, SD = 1.1, n = 20), 2.5–3.0 µm wide (x̅ = 2.5 μm, SD = 1.4, n = 20), globose to subglobose, aseptate and hyaline to subhyaline.

Also see Fernández et al. (1999), Réblová et al. (2016b) and Luo et al. (2019).

#### Notes

This species was originally collected on decaying wood in the Caribbean national forest and described as *Melanopsammellagonytrichii* F.A. Fernández & Huhndorf (Fernández and Huhndorf 2005), but later it was renamed as *Chloridiumgonytrichii* by Réblová et al. (2016b).

### Dictyocheirospora rotunda

M.J. D'souza, Bhat & K.D. Hyde, Fungal Diversity: 80(1), 457-482

34C69934-3D95-5BC7-9014-6CC973A77FDD

551581

#### Materials

- **Type status:** Other material. **Occurrence:** recordedBy: Chun-Sheng Long, Qi-Rui Li & Jian Ma; occurrenceID: EB9BB9B1-B71A-54A2-93F3-392E6A6859EC; **Taxon:** scientificName: *Dictyocheirosporarotunda*; **Location:** continent: Asia; country: China; stateProvince: Guizhou; county: Libo county; locality: Lanting Mountain Forest Park; verbatimElevation: 850 m; verbatimCoordinates: 25.1206N, 107.3298E; **Identification:** identifiedBy: Chun-Sheng Long; **Event:** eventTime: 11/21/2021; **Record Level:** collectionID: GMB040

#### Description

Conidiophores 3–5 μm long (x̅ = 2.8 μm, SD = 0.5, n = 20), 19.5–22.5 μm wide (x̅ = 3.6 μm, SD = 0.5, n = 20), micronematous, pale brown, smooth. Conidiogenous cells holoblastic, integrated, terminal, pale brown, cylindrical, smooth-walled. Conidia 49–55 μm long (x̅ = 52 μm, SD = 52.6, n = 20), 19.5–22.5 μm wide (x̅ = 21 μm, SD = 2.4, n = 20), solitary, acrogenous, cheiroid, pale brown to brown, consisting of 5–7 rows of cells, rows digitate, cylindrical, inwardly curved at the tip, arising from a basal cell euseptate, guttulate.

Also see Phukhamsakda et al. (2020).

#### Notes

*Dictyocheirosporarotunda*, the type species of *Dictyocheirospora*, was collected on submerged wood in freshwater from Thailand (Boonmee et al. 2016). It has been reported in Guizhou, China (Yang et al. 2018). *Dictyocheirosporarotunda* is similar to *D.heptaspora* (Garov.) M.J. D'souza, Boonmee & K.D. Hyde in morphology, but the rows of *D.rotunda* are not separable without manual force, whereas those of *D.heptaspora* are easily separable (Boonmee et al. 2016).

### Diplococcium dendrocalami

Goh, K.D. Hyde & Umali, Mycologia 90(3): 515 (1998)

E1B0DDBC-9A1B-5A4C-A5DF-6FB846046083

443599

#### Materials

- **Type status:** Other material. **Occurrence:** recordedBy: Chun-Sheng Long; occurrenceID: 5BA2D587-84EB-502E-B734-32CCAC974E59; **Taxon:** scientificName: *Diplococciumdendrocalami*; **Location:** continent: Asia; country: China; stateProvince: Guizhou; county: Guiyang; locality: Guizhou Medical University Campus; verbatimElevation: 1199 m; verbatimCoordinates: 26.5921N, 106.7143E; **Identification:** identifiedBy: Chun-Sheng Long, Qi-Rui Li & Jian Ma; **Event:** eventDate: 15/9/2021; habitat: On decaying wood; **Record Level:** collectionID: GMB0404

#### Description

Conidiophores 211–345.6 μm long (x̅ = 52.3 μm, SD = 52.3 n = 20), 4.4–9.5 µm wide (x̅ = 7.1 μm, SD = 2.1, n = 20), unbranched, erect, straight, attenuated, distinctly 5-8-septate, thick-walled, medium yellowish-brown, uniform in colour. Conidiogenous cells 120–280 μm long (x̅ = 199.1 μm, SD = 52.9, n = 20), 10–12 µm wide (x̅ = 10.8 μm, SD = 1.8, n = 20), integrated, polytretic with pores 0.8–1 μm diam., terminal and intercalary. Conidia 49–55 μm long (x̅ = 52 μm, SD = 5.2, n = 20), 19.5–22.5 μm wide (x̅ = 21 μm, SD = 3, n = 20), solitary, acrogenous, cheiroid, pale brown to brown, consisting of 5–7 rows of cells, rows digitate, cylindrical, inwardly curved at the tip, arising from a basal cell, without appendages, with each row composed of 8–12 cells, euseptate, guttulate, slightly constricted at septa.

Also see Goh et al. (1998) and Xia et al. (2017).

#### Notes

*Diplococciumdendrocalami* was firstly introduced in the culms of *Dendrocalamus* sp. in the Philippines (Goh et al. 1998) and later was found in Chongqing, China (Xia et al. 2017). *Diplococciumdendrocalami* is similar to *D.clavariarum* (Desm.) Hol.-Jech in morphology, but the conidiophores of *D.clavariarum* are branched and slender (10–12 µm vs. 3.5–6 µm) (Goh et al. 1998).

### Endophragmiella curvata

(Corda) S. Hughes, New Zeal. J. Bot. 17(2): 148(1979)

413862FA-4DA9-5532-8CCD-09A572A91842

313581

#### Materials

- **Type status:** Other material. **Occurrence:** recordedBy: Chun-Sheng Long; occurrenceID: A160A11E-BDCF-5472-B720-D322C6180316; **Taxon:** scientificName: *Endophragmiellacurvata*; **Location:** continent: Asia; country: China; stateProvince: Guizhou; county: Guiyang; locality: Guiyang Forest Park; verbatimElevation: 1190 m; verbatimCoordinates: 26.5702N, 106.7108E; **Identification:** identifiedBy: Chun-Sheng Long, Qi-Rui Li & Jian Ma; **Event:** eventDate: 25/9/2021; habitat: On decaying wood; **Record Level:** collectionID: GMB0407

#### Description

Conidiophores 37–66 μm long (x̅ = 52 μm, SD = 11.3, n = 20), 2.5–4.8 µm wide (x̅ = 3.1 μm, SD = 0.7, n = 20), macronematous, mononematous, single, unbranched, erect, straight or flexuous, septate, smooth, brown. Conidiogenous cells 3.6–5.6 μm long (x̅ = 4.4 μm, SD = 0.3, n = 20), 2.5–4.8 µm wide (x̅ = 3.6 μm, SD = 0.3, n = 20), monoblastic, integrated, terminal, cylindrical, smooth, brown to pale brown. Conidia 14.5–21 μm long (x̅ = 17.8 μm, SD = 3.3, n = 20), 6–7.5 µm wide (x̅ = 6.7 μm, SD = 0.5, n = 20), holoblastic, solitary, acrogenous, dry, clavate, smooth, lower two cells brown, apical cell pale brown, 2-septate.

Also see Hughes (1979) and Ma (2012).

#### Notes

*Endophragmiellacurvata* has been found on dead branches from Guangdong Province, China (Ma 2012). Morphologically, *E.curvata* is similar to *E.novae-zelandiae* S. Hughes (Hughes 1979), but the conidia of *E.novae-zelandiae* are larger than those of *E.curvata* (27–40 × 9.3–12.6 µm vs.14.5–21 × 6–7.5 µm) and the *E.novae-zelandiae* also has two septa conidia (Hughes 1979).

### Hemicorynespora clavata

(Corda) S. Hughes, New Zeal. J. Bot. 17(2): 148(1979)

FC3345F4-9000-5AD7-8B78-A08D1D525C21

510760

#### Materials

- **Type status:** Other material. **Occurrence:** recordedBy: Chun-Sheng Long; occurrenceID: 93225A11-2AB4-5DAB-900A-620C60B6C12A; **Taxon:** scientificName: *Hemicorynesporaclavata*; **Location:** continent: Asia; country: China; stateProvince: Guizhou; municipality: Guiyang; locality: Guizhou Medical University Campus; verbatimElevation: 1123 m; verbatimCoordinates: 26.5231N, 106.7163E; **Identification:** identifiedBy: Chun-Sheng Long, Qi-Rui Li & Jian Ma; **Event:** eventTime: 15/9/2021; habitat: On decaying wood; **Record Level:** collectionID: GMB0405

#### Description

Conidiophores 110–140 μm long (x̅ = 127.9 μm, SD =10, n = 20), 2.5–4.5 µm wide (x̅ = 2.9 μm, SD = 1.9, n = 20), macronematous, mononematous, single, unbranched, erect, straight or flexuous, septate, smooth, brown. Conidiogenous cells 12–13 μm long (x̅ = 12.4 μm, SD =0.34, n = 20), 3–4 µm wide (x̅ = 2.9 μm, SD = 0.4, n = 20), monoblastic, integrated, terminal, cylindrical, smooth, brown, percurrently proliferating. Conidia 12–17 μm long (x̅ = 14.1 μm, SD = 2.8, n = 20), 2–5 µm wide (x̅ = 3.4 μm, SD = 1.1, n = 20) holoblastic, solitary, acrogenous, dry, clavate, smooth, lower two cells brown, apical cell pale brown, 2–septate.

Also see Delgado et al. (2007).

#### Notes

Delgado et al. (2007) originally described this species on the stems of dead liana in Cuba and later Ma (2012) discovered it on dead branches in China. It superficially resembles *Hemicorynesporafusispora*, but the latter has spindle to inverted rods and longer conidia (12–20 µm vs. 15–30 µm) and its conidiogenous cells are shorter than those of *H.clavata* (12.5–21 vs. 15–18 µm) (Delgado et al. 2007).

### Kylindria excentrica

Bhat & B. Sutton, Trans. Br. mycol. Soc. 84(4): 728 (1985)

6B43901E-2635-52D8-9892-8DABAA3082B5

105413

#### Materials

- **Type status:** Other material. **Occurrence:** recordedBy: Chun-Sheng Long; occurrenceID: C1A8F2EF-0EC1-5EDB-B7AD-6279AD758FF5; **Taxon:** scientificName: *Kylindriaexcentrica*; **Location:** continent: Asia; country: China; stateProvince: Guizhou; county: Libo county; verbatimElevation: 875 m; verbatimCoordinates: 25.1205N, 107.3634E; **Identification:** identifiedBy: Chun-Sheng Long, Qi-Rui Li & Jian Ma; **Event:** eventDate: 11/21/2021; habitat: on decaying wood; **Record Level:** collectionID: GMB0398

#### Description

Conidiophores 200–350 μm long (x̅ = 280.6 μm, SD = 45, n = 20), 7–10 µm wide (x̅ = 8.6 μm, SD = 1.9, n = 20), mononematous, erect, simple, straight or flexuous, thick-walled, dark brown, paler towards the apex, 8–10 septate. Conidiogenous cells 48.5–60 μm long (x̅ = 53.5 μm, SD = 3.9, n = 20), 9–12 µm wide (x̅ = 10.2 μm, SD = 1.3, n = 20), with a narrow cytoplasmic channel and marked periclinal thickening in the upper quarter, lacking a collarette, proliferating enteroblastically to produce successive conidia at the same level. Conidia 21.5–40 μm long (x̅ = 29.3 μm, SD = 6.8, n = 20), 7.5–10 µm wide (x̅ = 8.2 μm, SD = 0.5, n = 20), holoblastic, solitary, accumulating in translucent slimy masses at the apices of conidiogenous, cylindrical, obtuse at the apex, slightly tapered towards the truncate base, hyaline, 3–euseptate, smooth, eguttulate.

Also see Bhat and Sutton (1985) and Xia et al. (2013).

#### Notes

*Kylindriaexcentrica* was firstly found on rotten wood in Ethiopia (Bhat and Sutton 1985). *Kylindriaexcentrica* is similar to *K.millettiae* Y.D. Zhang & X.G. Zhang in morphology, but differs markedly in conidial dimensions (19.5–24 × 6.5–9 μm vs. 27.5–35 × 7.5–8.5 μm) (Zhang et al. 2010). In addition, *K.excentrica* has a lateral flat scar in the conidial base, whereas those of *K.millettiae* have a lateral flat scar in the excentric (Zhang et al. 2010).

### Neohelicosporium griseum

(Berk. & M.A. Curtis) Y.Z. Lu & K.D. Hyde, Fungal Diversity 92: 241 (2018)

3C8897B8-A720-59A1-9811-43991C7587DB

GMB0412

#### Materials

- **Type status:** Holotype. **Occurrence:** recordedBy: Chun-Sheng Long; occurrenceID: 308A8DB4-3FA1-502F-B26E-7F5F12FE9F90; **Taxon:** scientificName: *Neohelicosporiumgriseum*; **Location:** continent: Asia; country: China; stateProvince: Yunnan; county: Nanjian Yi Autonomous County; locality: Lingbaoshan National Forest Park; verbatimElevation: 2418 m; verbatimCoordinates: 24.7342N, 100.4234E; **Identification:** identifiedBy: Chun-Sheng Long, Qi-Rui Li & Jian Ma; **Event:** eventTime: 18/8/2021; habitat: on decaying wood; **Record Level:** collectionID: GMB0412

#### Description

Conidiophores 3.5–4 µm diam. (x̅ = 3.6 μm, SD =1.2, n = 20), arising from a dark repent mycelium, more or less erect, dark brown, septate, irregularly branched, often forming a loop and network by anastomosing. Conidiogenous cells 1–1.5 µm long (x̅ = 1.3 μm, SD = 0.6, n = 20), 0.5–1 µm wide (x̅ = 0.8 μm, SD = 0.5, n = 20), holoblastic, monoblastic, integrated, intercalary or terminal, denticulate; denticles on the lower parts of conidiophores or directly arising on lateral of creeping fertile hyphae. Conidia diameter of coiled spores 12–15 µm (x̅ = 14.1 μm, SD =1.9, n = 20), pleurogenous, borne singly on minute hyaline sporogenous teeth, hyaline, tightly coiled 2½–3¼ times, indistinctly 18-20 septate.

Also see Lu et al. (2018).

#### Notes

Goos (1989) classified *Helicosporiumcinereum* Peck, *H.leptosporum* Sacc. and *H.lumbricoides* Sacc. as *H.griseum. Lu et al. (2018)* placed *H.griseum* and *H.lumbricoides* in *Neohelicosporium* Y.Z. Lu, J.C. Kang & K.D. Hyde as *N.griseum*, based on phylogenetic analysis. *Neohelicosporiumovoideum* Y.Z. Lu et al. and *N.griseum* are similar in morphology, but the conidium of *N.ovoideum* has fewer curls (3–4 vs. 2–3) (Lu et al. 2018).

### Phaeoisaria guttulata

J. Yang & K.D. Hyde, Mycosphere 9(2): 401 (2018)

E2E36275-A22F-5D66-8547-F60FECF83644

554233

#### Materials

- **Type status:** Holotype. **Occurrence:** occurrenceID: 2041B2E4-5BBA-5C4F-8136-70A7BEDE99A4; **Taxon:** scientificName: *Phaeoisariaguttulata*; **Location:** continent: Asia; country: China; stateProvince: Guizhou; county: Sandu Autonomous County; locality: Yaorenshan National Forest Park; verbatimElevation: 632 m; verbatimCoordinates: 26.5535N, 106.7533E; **Identification:** identifiedBy: Chun-Sheng Long, Qi-Rui Li & Jian Ma; **Event:** eventDate: 9/9/2021; habitat: on decaying wood; **Record Level:** collectionID: GMB0402

#### Description

Conidiophores 480–520 μm long (x̅ = 280.6 μm, SD =4 5, n = 20), 2–5 µm wide (x̅ = 3.7 μm, SD = 1.3, n = 20) macronematous, synnematous, erect, septate, smooth, mid-brown to dark brown. Conidiogenous cells 14.5–35.9 μm long (x̅ = 26.6 μm, SD = 4.4, n = 20), 1.6–3.8 μm wide (x̅ = 3.0 μm, SD = 0.6, n = 20) integrated, terminal, polyblastic, pale brown to hyaline, sympodial, splaying out with one to several denticulate conidiogenous cells loci. Conidia 3.5–5.5 μm long (x̅ = 4.5 μm, SD =1.1, n = 20), 2.5–4.8 µm wide (x̅ = 3.5 μm, SD =1.4, n = 20), globose to obovoid, hyaline, smooth-walled, guttulate, aseptate.

Also see Hyde et al. (2018).

#### Notes

This species was originally discovered on decaying wood in Guizhou Province, China (Hyde et al. 2018). It is similar to *P.clavulata* (Grove) E. W. Mason & S. Hughes in conidial shape, but the latter has smaller globose conidia (3.5–5.5 µm vs. 1–2 µm) (Révay 1985, Hyde et al. 2018).

### Phragmocephala atra

(Berk. & Broome) E.W. Mason & S. Hughes, Naturalist: 97 (1951)

496EB05A-0F41-593E-B37F-D2ACEC8C34C8

303243

#### Materials

- **Type status:** Holotype. **Occurrence:** recordedBy: Chun-Sheng Long; occurrenceID: 4FC3FA5F-FE62-5C51-8700-C051B063DE42; **Taxon:** scientificName: *Phragmocephalaatra*; **Location:** continent: Asia; country: China; stateProvince: Guizhou; municipality: Guiyang; locality: Guiyang Forest Park; verbatimElevation: 1187 m; verbatimCoordinates: 26.5723N, 106.7432E; **Identification:** identifiedBy: Chun-Sheng Long, Qi-Rui Li & Jian Ma; **Event:** eventTime: 9/9/2021; habitat: On decaying wood; **Record Level:** collectionID: GMB0406

#### Description

Conidiophores 128–157 μm long (x̄ = 142.5 µm, SD = 14.5, n = 20), 6.5–8.5 μm wide (x̄ = 7.5 µm, SD = 1, n = 20), synnematous, macronematous, septate, unbranched or branched, erect, dark brown at the base, pale brown at fertile, flared apex, sometimes proliferating, 5–8-septate. Conidiogenous cells 37–44 μm long (x̄ = 37.3 µm, SD = 2.1 µm, n = 20), 2.3–4 μm wide (x̄ = 2.5 µm, SD = 1.3 µm, n = 20), monoblastic, terminal, integrated, elongated, pale brown, often separating from the conidium through a break or frill below the base of conidium. Conidia 30–35 μm long (x̄ = 32.5 µm, SD = 2.5 µm, n = 20), 16–19 μm wide (x̄ = 17.5 µm, SD = 1.5 µm, n = 20), 4–septate, ellipsoidal to subglobose, dark brown, pale brown at apical and basal cells, with dark brown to black central cells, with a thick dark band on the central septum; smooth, rounded at apex, truncate at base, sometimes released with part of conidiogenous cell.

Also see Mason and Hughes (1951).

#### Notes

*Phragmocephalaatra* is the type species of *Phragmocephala*, which is characterised by the dark brown to black central cells (Mason and Hughes 1951, Su et al. 2015). *Phragmocephalaatra* has been reported in Yunnan Province, China (Su et al. 2015).

### Cryptophiale udagawae

Piroz. & Ichinoe, Can. J. Bot. 46: 1126 (1968)

5AB07660-80D7-579A-97F7-E062482AA7E6

329371

#### Materials

- **Type status:** Other material. **Occurrence:** recordedBy: Chun-Sheng Long; occurrenceID: F7EFF95D-CA2D-566D-A743-FAC90D454F35; **Taxon:** scientificName: *Cryptophialeudagawae*; **Location:** continent: Asia; country: China; stateProvince: Guizhou; county: Luodian County; locality: Hongshui river; verbatimElevation: 399 m; verbatimCoordinates: 25.2239N, 106.5349E; **Identification:** identifiedBy: Chun-Sheng Long, Qi-Rui Li & Jian Ma; **Event:** eventDate: 18/9/2021; habitat: on decaying wood; **Record Level:** collectionID: GMB0408

#### Description

Conidiophores 97–120 μm long (x̄ = 99.7 µm, SD = 8.3, n = 20), 4.5–9 μm wide (x̄ = 6.2 µm, SD = 1.3, n = 20), straight or flexuous, septate, smooth, brown, with 3-4 branches at the apex. Conidiogenous cells 39–46 μm long (x̄ = 42.1 µm, SD = 4.0, n = 20), 7.6–12 μm wide (x̄ = 8.7 µm, SD = 2.4, n = 20) enteroblastic, phialidic, obscured by a shield of sterile cells. Conidia solitary, 1–septate, falcate, simple, smooth, hyaline, produced in slimy masses, 15.5–18 μm long (x̄ = 16.4 µm, SD = 1, n = 20), 1.2–1.4 μm wide (x̄ =1.3 µm, SD = 1.1, n = 20), solitary, 1–septate, falcate, simple, smooth, hyaline, produced in slimy masses.

Also see Pirozynski (1968).

#### Notes

Pirozynski (1968) described the species from fallen leaves in Japan. Ma et al. (2010) and Yang et al. (2019) discovered the species in China. *Cryptophialeudagawae* shows a variable number of branches in the conidiophore. There are 1–3 branches on *C.udagawae* in Pirozynski (1968), three in Matsushima (1971), 5–8 in Mercado-Sierra et al. (1997) and three in our specimen.

### Ellisembia brachypus

(Ellis & Everh.) Subram., Proc. Indian natn Sci. Acad., Part B. Biol. Sci. 58(4): 183 (1992)

C30FE46E-7BB6-50EB-B0D3-9D29E1834254

306280

#### Materials

- **Type status:** Other material. **Occurrence:** recordedBy: Chun-Sheng Long; occurrenceID: 8AE6F3F9-5C0F-533E-BB66-C60F6DECAD67; **Taxon:** scientificName: *Ellisembiabrachypus*; **Location:** continent: Asia; country: China; stateProvince: Guizhou; municipality: Guiyang; locality: Guizhou Medical University Campus; verbatimElevation: 1217 m; verbatimCoordinates: 26.5943°N, 106.734513°E; **Identification:** identifiedBy: Chun-Sheng Long, Qi-Rui Li & Jian Ma; **Event:** eventDate: 15/9/2021; habitat: on decaying woo; **Record Level:** collectionID: GMB0401

#### Description

Conidiophores 86–114 μm long (x̄ = 100 µm, SD = 5.3, n = 20), 5–7 μm wide (x̄ = 100 µm, SD = 5.3, n = 20) macronematous, mononematous, solitary, erect, unbranched, 7–9 septate, straight or flexuous, percurrently growing, dark brown, smooth. Conidiogenous cells 5–7 μm long (x̄ = 5.3 µm, SD = 2.2, n = 20), 4–5 μm long (x̄ = 4.8 µm, SD = 2.4, n = 20), monoblastic, integrated, terminal, dark brown. Conidia 45–63 μm long (x̄ = 54 µm, SD = 5.3, n = 20), 13–17 μm wide (x̄ = 15 µm, SD = 2.3, n = 20), acrogenous, solitary, ovoid to fusiform, 5–6-pseudoseptate, truncate at base, with a short and hyaline rostrate tip at apex, brown, smooth-walled.

Also see Hughes (1958) and Luo et al. (2019).

#### Notes

*Ellisembiabrachypus* was firstly reported as *Sporidesmiumbranchypus* in Shiwaliks (Prasher and Singh 2014) and previously collected on dead branches of *Moringaaoleifera* Lam. in Kerela and Rajasthan (Bilgrami et al. 1991, Jamaludeen et al. 2004). Later, it was found in Yunnan, China (Luo et al. 2019).

### Vanakripa menglensis

D.M. Hu, L. Cai, K.D. Hyde, Sydowia 62(2): 199(2010)

0FEF0D72-1470-5606-BD3D-6A5B4D6D4CD7

518634

#### Materials

- **Type status:** Other material. **Occurrence:** recordedBy: Chun-Sheng Long; occurrenceID: 609FEB39-B6F6-59E8-B300-07FE401FC962; **Taxon:** scientificName: *Vanakripamenglensis*; **Location:** continent: Asia; country: China; stateProvince: Yunnan; county: Nanjian Yi Autonomous County; locality: Lingbaoshan National Forest Park; verbatimElevation: 2231 m; verbatimCoordinates: 24.7861N, 100.4846E; **Identification:** identifiedBy: Chun-Sheng Long, Qi-Rui Li & Jian Ma; **Event:** eventDate: 18/8/2021; habitat: on decaying wood; **Record Level:** collectionID: GMB0411

#### Description

Conidiophores 7.5–8.3 μm long (x̄ =7.9 µm, SD = 3.2, n = 20), 2.5–3 µm wide (x̄ = 2.6 µm, SD = 2, n = 20), micronematous, hypha-like, cylindrical, aseptate, simple or sparsely branched, smooth, hyaline. Conidiogenous cells 20–40 μm long (x̄ =34.2 µm, SD = 4.3, n = 20), 4–6 µm wide (x̄ = 5.3 µm, SD = 2.3, n = 20), hyaline, clavate to vermiform. Conidia 17–23 μm long (x̄ = 20.4 µm, SD = 4.1, n = 20), 8–13 µm wide (x̄ = 11.3 µm, SD = 3.3, n = 20), acrogenous, solitary, clavate to obpyriform, smooth, brown to dark brown, aseptate.

Also see Hu et al. (2010).

#### Notes

The genus *Vanakripa* was originally established by Bhat and Kendrick (1993). So far, ten epithets for *Vanakripa* are listed in Index Fungorum (1 June 2022). *Vanakripamenglensis* is distinguished by its clavate to obpyriform conidia (Hu et al. 2010). *V.menglensis* has been reported from Yunnan Province, China (Hu et al. 2010).

### Sporidesmium conversum

W.P. Wu, Fungal Diversity Res. Ser. 15: 27(2005).

1033200B-BEC1-539D-8330-C365BA5EBE92

356332

#### Materials

- **Type status:** Holotype. **Occurrence:** recordedBy: Chun-Sheng Long; occurrenceID: B43EED21-3E28-5941-9C29-64A4C7DFA7C8; **Taxon:** scientificName: *Sporidesmiumconversum*; **Location:** continent: Asia; country: China; stateProvince: Guizhou; municipality: Guiyang; locality: Guizhou Medical University Campus; verbatimElevation: 1207 m; verbatimCoordinates: 26.3967N, 106.7161E; **Identification:** identifiedBy: ChunSheng Long, Qi-Rui Li & Jian Ma; **Event:** eventDate: 15/9/2021; habitat: on decaying wood; **Record Level:** collectionID: GMB0403

#### Description

Conidiophores 37–50 μm long (x̄ = 42.2 µm, SD = 4.6, n = 20), 5–6.8 µm wide (x̄ = 5.9 µm, SD = 0.6, n = 20), macronematous, mononematous, solitary, erect, unbranched, 1–3 septate. Conidiogenous cells 10–12 μm long (x̄ =10.8 µm, SD =0.5, n = 20), 4-5 μm long (x̄ = 4.8 µm, SD = 2.4, n = 20), monoblastic, integrated, terminal, dark brown. Conidia 36–52 μm long (x̄ = 36.6 µm, SD = 12.7, n = 20), 8–10 μm wide (x̄ = 9.4 µm, SD = 2.5, n = 20), acrogenous, solitary, clavate to broadly fusiform, 5–6 septate, with a short and hyaline rostrate tip at apex, brown, smooth-walled.

Also see Wu (2005).

#### Notes

Wu (2005) firstly described this specis from China. Species similar to *S.conversum*, that produce conidia with barrel- to ampoule-shaped layers and inverted rod to spindle-shaped conidia include *S.australiense* M.B. Ellis, *S.clarki* P.M. Kirk, *S.hamatum* M.B. Ellis, *S.pedunculatum* (Peck) M.B. Ellis, *S.rubi* M. B. Ellis and *S.uapacae* M.B. Ellis (Wu and Zhuang 2008). The difference between *S.conversum* and all these species is that the conidial apex in *S.conversum* has cap-like and conical mucinous appendages (Wu and Zhuang 2008).

## Discussion

Many genera of hyphomycetes were found in Karst areas, such as *Acrogenospora* M.B. Ellis, *Craspedodidymum* Hol.-Jech, *Corynesporopsis* P.M. Kirk, *Dactylella* Grove, *Dendryphiopsis* S. Hughes, *Digitoramispora* R.F. Castañeda & W.B. Kendr., *Diplocladiella* G. Arnaud, *Endophragmiella* B. Sutton, *Elegantimyces* Goh, C.K.M. Tsui & K.D. Hyde, *Exosporium* Link, *Gangliostilbe* Subram. & Vittal, *Helminthosporium* Link, *Heteroconium* Petr., *Microclava* F. Stevens, *Monodictys* S. Hughes, *Mucispora* Jing Yang, Bhat & K.D.Hyde, *Phalangispora* Nawawi & J. Webster, *Phragmocephala* E.W. Mason & S. Hughes, *Repetophragma* Subram., *Spadicoides* S. Hughes, *Sympodioplanus* R.C. Sinclair & Boshoff, *Synnemacrodictys* W.A. Baker & Morgan-Jones, *Tretospeira* Piroz. and *Ulocladium* Preuss (Gao et al. 1997, Zhao and Li 1997, Wang et al. 2008, Yang et al. 2012, Li et al. 2017, Li et al. 2014, Li et al. 2019, Guo et al. 2019). Through the investigation of Zhang (2012), 4000 samples were collected in China including Fujian, Guangdong, Guangxi, Guizhou, Hainan, Hunan, Sichuan and Yunnan Provinces. Here, nineteen species on decaying wood were recorded from south China including Karst areas. However, after many attempts, we only obtained a very small amount of pure cultures.

After consulting the relevant literature and our experimental experience, the authors found that numerous hyphomycetes occurring on wood cannot be cultured which led to lack of DNA sequences, resulting in confusion in identification and classification (Réblová et al. 2016a). For example, *Chloridiumgonytrichii* was originally considered as the genus *Melanopsammella*, based on its morphology (Fernández and Huhndorf 2005). However, Crous et al. (2012) confirmed that *M.gonytrichii* was more closely related to *Chloridium* according to its phylogenetic analyses. Most conidia are difficult to germinate on artificial medium (Rousseau and Halvorson 1969, Zhu et al. 2016, Wijayawardene et al. 2021). Therefore, the identification of hyphomycetes, based on DNA sequences, is limited (Zhang 2018). Direct DNA extraction would be the solution (Wijayawardene et al. 2021). However, there are also some problems with direct DNA extraction methods. The requirements for picking a small number of conidia or *mycelium* are relatively high. For some species with smaller conidia and fewer hyphae on the substrate, especially those with colourless or hyaline conidia, it is difficult to obtain a sufficient amount of material for direct DNA extraction. Moreover, it is often difficult to amplify the protein gene when the directly extracted DNA is amplified by PCR.

## Supplementary Material

XML Treatment for Coryneum septemseptatum

XML Treatment for Phaeoisaria guizhouensis

XML Treatment for Pleurothecium yunanensis

XML Treatment for Brachysporiella pulchra

XML Treatment for Brachysporiella setosa

XML Treatment for Catenularia catenulata

XML Treatment for Chloridium gonytrichii

XML Treatment for Dictyocheirospora rotunda

XML Treatment for Diplococcium dendrocalami

XML Treatment for Endophragmiella curvata

XML Treatment for Hemicorynespora clavata

XML Treatment for Kylindria excentrica

XML Treatment for Neohelicosporium griseum

XML Treatment for Phaeoisaria guttulata

XML Treatment for Phragmocephala atra

XML Treatment for Cryptophiale udagawae

XML Treatment for Ellisembia brachypus

XML Treatment for Vanakripa menglensis

XML Treatment for Sporidesmium conversum

## Figures and Tables

**Table 1.** Strains used in the phylogenetic tree and their culture accession and GenBank numbers. Strains from this study are in bold.

**SPECIES**	**Strain number**		**GenBank Accession number**		
			**ITS**	**LSU**	**tef**
*Asterosporiumasterospermum*	KT2125		NA	AB553743	NA
*Asterosporiumasterospermum*	KT2138		NA	AB553744	NA
*Chaetoconispolygoni*	CBS 405.95		NA	EU754141	NA
*Coryneumcastaneicola*	CFCC 52315		MH683559	MH683551	MH685731
*Coryneumcastaneicola*	CFCC 52316		MH683560	MH683552	MH685732
*Coryneumdepressum*	AR 3897		NA	EU683074	NA
*Coryneumgigasporum*	G14		MK799957	MK799944	MK799830
*Coryneumgigasporum*	G15		MK799958	MK799945	MK799831
*Coryneumheveanum*	MFLUCC 17-0369		MH778707	MH778703	NA
*Coryneumheveanum*	MFLUCC 17-0376		MH778708	MH778704	MH780882
*Coryneumilicis*	CFCC 52994		MK799948	MK799935	NA
*Coryneumilicis*	CFCC 52995		MK799949	MK799936	NA
*Coryneummodonia*	AR 3558		NA	EU683073	NA
*Coryneumperniciosum*	CBS 130.25		MH854812	MH866313	NA
*Coryneumsinense*	X60	MK799952		MK799939	MK799825
*Coryneumsinense*	X23	MK799953		MK799940	MK799826
*Coryneumseptemseptatum*	GMB0393	OQ540748		OQ540743	OQ540767
*Coryneumseptemseptatum*	GMB0392	OQ560328		OQ560329	NA
*Coryneumsongshanense*	CFCC 52997	MK799946		MK799933	MK799822
*Coryneumsongshanense*	CFCC 52998	MK799947		MK799934	MK799823
*Coryneumsuttonii*	Z17	MK799955		MK799942	MK799828
*Coryneumsuttonii*	Z63	MK799956		MK799943	MK799829
*Coryneumumbonatum*	CBS 199.68		MH859114	MH870828	NA
*Coryneumumbonatum*	AR 3541		NA	EU683072	NA
*Crinitosporapulchra*	CPC 22807		KJ710466	KJ710443	NA
*Cytosporacentrivillosa*	IT 2132		MF190122	MF190068	NA
*Cytosporacentrivillosa*	MFLU 17-0887		MF190123	MF190069	NA
*Cytosporafertilis*	AR 3514		NA	EU255210	EU222018
*Cytosporamelanodiscus*	Jimslanding2		JX438621	NA	JX438605
*Cytosporatranslucens*	CZ320		FJ755269	FJ755269	NA
*Diaportheazadirachtae*	TN 01		KC631323	NA	NA
*Diaportheeres*	AR 5193		KJ210529	MT378367	KJ210550
*Diaportheeres*	T-098		MF190138	MF190081	MF377595
*Diaporthemaytenicola*	CPC 21896		KF777157	KF777210	NA
*Hyaliappendisporagalii*	MFLU 15-2269		MF190150	MF190095	MF377587
*Lamproconiumdesmazieri*	MFLUCC 15-0870		KX430134	KX430135	MF377591
*Lamproconiumdesmazieri*	MFLUCC 15-0872		KX430138	KX430139	MF377593
*Macrohilumeucalypti*	CPC 10945		DQ195781	DQ195793	NA
*Macrohilumeucalypti*	CPC 19421		KR873244	KR873275	NA
*Pachytrypeprinceps*	Rogers s.n.		NA	FJ532382	NA
*Pachytryperimosa*	FF1066		NA	FJ532381	NA
*Phaeoacremoniumaleophilum*	CBS 631.94		AF266647	AB278175	KF764643
*Phaeoacremoniumvibratile*	CBS 117115		KF764573	DQ649065	KF764645
*Phaeoappendicosporathailandensis*	TL 19		MF190157	MF190102	NA
*Phaeoappendicosporathailandensis*	MFLU 12-2131		MF190158	MF190103	NA
*Phaeodiaportheappendiculata*	D77		KF570156	KF570156	NA
*Prosopidicolamexicana*	CBS 113529		AY720709	KX228354	NA
*Prosopidicolamexicana*	CBS 113530		AY720710	NA	NA
*Rossmaniaukurunduensis*	AR 3484		NA	EU683075	NA
*Stegonsporiumacerophilum*	D5		EU039982	EU039993	EU040027
*Stegonsporiumpyriforme*	D2		EU039971	EU039987	EU040001
*Stilbosporamacrosperma*	CBS 121883		JX517290	JX517299	KF570235
*Stilbosporamacrosperma*	WJ 1840		NA	AY616229	NA
*Sydowielladepressula*	CBS 813.79		NA	EU683077	NA
*Sydowiellafenestrans*	CBS 125530		JF681956	EU683078	MK524463

**Table 2.** Conidial sizes and numbers of distosepta of currently accepted *Coryneum* species.

Species name	Conidia size (μm)	No. of distosepta	Reference
*Coryneumacaciae*	49-52 × 5-6	5-6	Mcalpine (1903)
*Coryneumaffine*	20-22 x 7	7	Saccardo (1882)
*Coryneumarausiacum*	42–56 × 13–16	4–6	Senanayake et al. (2017)
*Coryneumbetulinum*	31–36 × 14–17	4–5	Sutton (1975)
*Coryneumberkeleyi*	30 x 8	3-5	Cooke (1906)
*Coryneumcalophylli*	38–48 × 12.5–14.5	5–6	Sutton (1975)
*Coryneumcanadense*	45-75 x 13-15	3-5	Bubak (1916)
*Coryneumcarpinicola*	50-68 x 8-11	7-11	Sutton (1975)
*Coryneumcastaneicola*	56–80 × 9.5–13	5-8	Sutton (1975)
*Coryneumclusiae*	30–40 × 20–30	3-5	Sutton (1975)
*Coryneumcompactum*	40–58 × 15–21	4-6	Sutton (1975)
*Coryneumcesatii*	80-90 x 13-15	6-7	Sutton (1975)
*Coryneumcocois*	40-42 x 3-4	2	Hennings (1903)
*Coryneumconcolor*	10-11 x 4.5-5	3	Penzig (1882)
*Coryneumdepressum*	44–53 × 19–23	4-6	Sutton (1975)
*Coryneumelevatum*	56-59 x 20-25	5-7	Sutton (1975)
*Coryneumeriobotryae*	5-9 x 5-7	1	Saccardo and Traverso (1906)
*Coryneumfagi*	45–75 × 10–15.5	6–12	Boonmee et al. (2021)
*Coryneumfoliorum*	15-20 x 6-8	3	Saccardo and Traverso (1902)
*Coryneumgigasporum*	93–108 × 19–21	7-9	Jiang et al. (2018)
*Coryneumgregoryi*	32.5–43 × 12–16	5-9	Sutton (1975)
*Coryneumheveanum*	43–53 x 15–20	4-6	Senwanna et al. (2018)
*Coryneumilicis*	82-105 × 9.5-12.5	10–11	Jiang et al. (2019)
*Coryneumjaponicum*	45–60 × 11–12	5-7	Sutton (1975)
*Coryneumlanciforme*	45–53 × 16–18	4-6	Sutton (1975)
*Coryneumlongistipitatum*	18-20 x 8-9	3	Saccardo and Traverso (1892)
*Coryneummegaspermum*	52-110 x 12-15	8-14	Theissen (1912)
Coryneummegaspermumvar.cylindricum	100–125 × 10–13	7-8	Sutton (1975)
*Coryneummodonium*	50–71 × 14–19	5-8	Sutton (1975)
*Coryneumneesii*	68–82 × 18–22	6–8	Sutton (1975)
*Coryneumpruni*	14–23 × 5.5–9	4–5	Wijayawardene et al. (2016)
*Coryneumpsidii*	25–40 × 14–17	5–6	Sutton (1975)
*Coryneumpyricola*	61–70 × 24–32	5–7	Sutton (1975)
*Coryneumseptemseptatum*	34–46 × 14.5–17	7–8	This study
*Coryneumquercinum*	45–60 × 14–16	6–7	Muthumary and Sutton (1986)
*Coryneumsinense*	50–76 × 13–17	5–7	Jiang et al. (2018)
*Coryneumsongshanense*	51–76 × 9–11.5	5–7	Jiang et al. (2019)
*Coryneumstromatoideum*	105–180 × 16–20	9–17	Sutton (1975)
*Coryneumsuttonii*	60–76 × 10–14.5	4–5	Jiang et al. (2018)
*Coryneumsydowianum*	50–58 × 14–17	5–6	Jiang et al. (2018)
*Coryneumumbonatum*	57–72 × 13–16	5–7	Sutton (1975)
